# Pickering emulsion as an oral delivery platform of anti-TNF-α antibody for ulcerative colitis therapy

**DOI:** 10.1016/j.ijpharm.2026.126881

**Published:** 2026-04-13

**Authors:** Jin Xie, Grahmm A. Funk, Su Jeong Song, Xiaodi Li, Connor S.E. Ahlquist, Andrea L. Villela-Nava, Hyunjoon Kim

**Affiliations:** a Department of Pharmaceutical Chemistry, University of Kansas, Lawrence, KS 66047, USA; b Bioengineering Program, University of Kansas, Lawrence, KS 66047, USA

**Keywords:** Pickering emulsion, Oral delivery, Anti-TNF-α antibody, Casein, Ulcerative colitis

## Abstract

Monoclonal antibodies that block pro-inflammatory cytokine tumor necrosis factor-alpha (TNF-α) are effective therapy for inflammatory bowel diseases (IBD). However, intravenous infusion (IV), the standard route of administration of anti-TNF-α monoclonal antibodies (TNF-Ab), is time-consuming and inconvenient for patients. Recently, subcutaneously administered TNF-Ab formulations were developed for at-home injections of maintenance doses, but self-injection using needle-based pen systems can lead to low patient compliance. Therefore, an oral TNF-Ab formulation could significantly increase the medication adherence and quality of life of IBD patients. In this work, we developed a patient-friendly formulation based on a casein-stabilized Pickering emulsion for oral delivery of TNF-Ab (Casein-PE@TNF-Ab). Pickering emulsion was employed to protect TNF-Ab from gastric degradation and enhance colon residence time. Casein-PE@TNF-Ab exhibited a uniform particle size of 300 nm, a zeta potential of 45 mV, and encapsulation efficiency above 80%. In vitro studies showed enhanced uptake of Casein-PE@TNF-Ab by lipopolysaccharide-stimulated RAW264.7 macrophages compared with non-stimulated cells. In vivo imaging demonstrated prolonged colonic retention relative to free TNF-Ab. In DSS-induced colitis mice, oral Casein-PE@TNF-Ab markedly increased colon length and suppressed colonic TNF-α, IL-1β, and MPO levels by 82%, 61%, and 62%, respectively, versus DSS. Neutrophil infiltration in the colon also decreased by 46% relative to DSS group. The formulation remained stable for 3 months at 4 °C with preserved particle size and TNF-α–neutralizing activity. Together, these findings indicate that casein-stabilized Pickering emulsions provide a promising oral platform for antibody delivery and represent a viable strategy for ulcerative colitis therapy.

## 1. Introduction

Ulcerative colitis (UC) is a chronic idiopathic form of inflammatory bowel disease (IBD) characterized by persistent inflammation at the colonic mucosa, which often leads to recurrent episodes of bloody diarrhea (Ordas et al., 2012). Although the exact etiology of UC remains unclear, it is widely accepted that a complex interplay among genetic predisposition, environmental triggers, and immune dysregulation contributes to disease onset and progression (Ananthakrishnan et al., 2018). The clinical management of UC remains challenging due to its relapsing nature and the difficulty in achieving sustained immune homeostasis. Current therapeutic approaches include 5-aminosalicylic acid (5-ASA), corticosteroids, immunosuppressants (e.g., 6-mercaptopurine, tacrolimus, mycophenolate mofetil), and biologic agents (e.g., infliximab, adalimumab, vedolizumab) (Raine et al., 2022). Among these, anti-tumor necrosis factor-alpha (anti-TNF-α) antibodies (TNF-Ab) are one of the standard treatments for patients with moderate to severe UC (Silva et al., 2010). Their therapeutic effects are mediated primarily through two mechanisms: neutralization of soluble TNF-α (sTNF-α) and interaction with transmembrane TNF-α (tmTNF-α), thereby suppressing pro-inflammatory signaling cascades and modulating immune responses (Palladino et al., 2003). Despite the promising clinical efficacy, TNF-Ab administered via intravenous (IV) and subcutaneous (S.C) routes, poses several limitations including systemic side effects, long infusion hours (in the case of IV), high treatment costs, and reduced patient compliance associated with frequent injections (Pugliese et al., 2017; Wang et al., 2015). Hence, oral delivery represents a promising alternative, offering the potential for improved patient adherence, reduced systemic exposure, and targeted drug release at the site of inflammation (A. des Rieux, V. Fievez, M. Garinot, Y.J. Schneider, V. Preat,, 2006).

Despite the potential advantages of oral antibody delivery, its clinical applicability remains limited by two principal challenges (Duran-Lobato et al., 2020; Hearnden et al., 2012). First, biological drugs including antibodies undergo gastrointestinal (GI) degradation during upper-GI transit due to gastric acidity and digestive proteases destabilizing and denaturing the protein structure, which markedly reduces the fraction that reaches inflamed colonic tissue and weakens therapeutic efficacy (Dos Santos et al., 2021). Second, colonic residence time of biologics is often insufficient in ulcerative colitis patients, as accelerated transit and frequent stools shorten luminal exposure of biological drugs at the inflamed sites (Chen et al., 2021; Haase et al., 2016). To address these barriers, innovative delivery approaches have been developed to preserve biological drugs’ activity during GI transit and facilitate targeted passage across the mucosal interface. These systems include nanocomplexes (Chen et al., 2023); nano-vesicles (Shen et al., 2020); and nanoparticles (Shrestha et al., 2022), which demonstrated enhanced intestinal permeability and targeted delivery to inflamed mucosal tissues.

Recently, Pickering emulsions have emerged as a promising platform for antibody delivery, owing to their unique structural and physicochemical properties. Unlike conventional emulsions stabilized by surfactants, Pickering emulsions are stabilized by solid particles irreversibly adsorbed at the oil–water interface, forming a robust interfacial layer (Du et al., 2024; Wu and Ma, 2016). This structural feature confers superior physical stability and resistance to coalescence, even under harsh conditions including those in the GI tract (Li et al., 2023). In addition, studies indicate that Pickering emulsions increase colonic residence by enhancing mucoadhesion, as their cohesive droplets adhere to colonic mucus and maintain prolonged contact, thereby sustaining local antibody exposure at inflamed sites (Mei et al., 2025; Niu et al., 2025; Wang et al., 2020). Our previous study demonstrated that polymeric nanoparticle stabilized Pickering emulsions can serve as an effective oral vaccine delivery system by protecting antigens from gastric degradation and enhancing their immunogenicity (Xie et al., 2025). These findings suggest that Pickering emulsions can be an effective oral delivery platform for biologics, including antibodies.

In this study, we developed a Pickering emulsion stabilized with casein, a milk-derived protein. Upon exposure to the acidic and enzyme-rich gastric environment, casein coagulates into a clot-like structure, leading to delayed gastric emptying and slower digestion (Boirie et al., 1997) (Thevenot et al., 2017). The resulting slow-digesting behavior hinders enzyme access and delays proteolysis, which makes casein a strong candidate for stabilizing oral delivery systems designed to protect labile biomacromolecules during GI transit. Taken together, these attributes suggest that a casein-based Pickering emulsion can address the two central barriers by protecting antibodies from gastric and enzymatic degradation and by prolonging colonic residence. In this study, a casein-stabilized Pickering emulsion was fabricated via a simple and reproducible method, exhibiting excellent physical stability. By leveraging the clot-forming properties of casein in the acidic gastric environment and the protective capability of Pickering emulsions for encapsulated biomacromolecules, this platform is expected to efficiently deliver TNF-Ab to the inflamed colon. Upon reaching the target site, the released antibody is anticipated to neutralize TNF-α, downregulate myeloperoxidase (MPO) expression, and suppress pro-inflammatory immune cell infiltration, thereby enhancing the therapeutic efficacy against UC.

## 2. Materials and methods

### 2.1. Materials

Squalene and casein were purchased from Sigma-Aldrich (St. Louis, MO, USA). Ovalbumin Alexa Fluor^™^, 647 Conjugate (OVA-647) was purchased from Invitrogen (Waltham, MA, USA), InVivoMAb anti-mouse TNF-α antibody was purchased from Bio X Cell. Flow cytometry antibodies were purchased from Biolegend (San Diego, CA).

### 2.2. Preparation of Casein-PE@TNF-Ab

The Pickering emulsion was prepared using a sonication method (Du et al., 2022). Briefly, 6 mg of casein was dissolved in PBS and mixed with 83 μL of squalene, which served as the oil phase. The mixture was sonicated at 90% amplitude for 2 min using a pulsed cycle of 6 s on and 1 s off (QSONICA Sonicator). Following sonication, TNF-α antibody stock solution was added to the emulsion and incubated at 4 °C with gentle shaking (100 rpm) for 1 h. For fluorescent tracking, OVA-647 was used in place of native OVA to prepare fluorescently labeled Casein-PE@OVA-647.

### 2.3. Characterization of Casein-PE@TNF-Ab

To optimize the Casein-PE@TNF-Ab, various concentrations of casein (2, 4, 6, 8, 10, and 12 mg/mL) were evaluated. Additionally, different water-to-oil ratios (8:1, 10:1, 12:1, 14:1, and 16:1) were tested to assess their effects on emulsion properties. The formulations were evaluated based on particle size, zeta potential, visual stability over a 7-day period, and TNF-Ab encapsulation efficiency, which served as the criteria for optimization. The particle size and zeta potential of Casein-PE@TNF-Ab were measured using dynamic light scattering (DLS) with a Zetasizer Nano ZS instrument (Malvern Instruments, UK). To evaluate formulation stability over time, samples were stored at 4 °C, and aliquots were collected on days 1, 3, 5, and 7 for analysis of particle size, zeta potential, and antibody encapsulation efficiency. **Encapsulation efficiency** – OVA-647 was used as a fluorescent surrogate for TNF-Ab during formulation optimization to prepare Casein-PE@OVA-647. The formulation was processed using Amicon^®^ Ultra centrifugal filters (50 kDa molecular weight cut-off) and centrifuged at 4000 rpm for 15 min to separate unencapsulated OVA-647. The fluorescence intensity of the filtrate was measured using a BioTek Cytation 7 plate reader (excitation: 650 nm; emission: 668 nm), and the concentration of free OVA-647 was determined using a standard calibration curve. For TNF-Ab quantification, Casein-PE@TNF-Ab was processed using Amicon^®^ Ultra centrifugal filters (300 kDa molecular weight cut-off) and centrifuged at 4000 rpm for 20 min to separate unencapsulated TNF-Ab. The amount of free TNF-Ab in the filtrate was quantified using ELISA kit according to the manufacturer’s instructions.

To analyze the structural characteristics of Casein-PE@TNF-Ab, Fourier transform infrared (FTIR) spectroscopy was performed using a Nicolet^™^ Summit^™^ FTIR Spectrometer. Samples of Casein-PE@TNF-Ab and TNF-Ab solution were lyophilized into powders using a freeze dryer (Labconco) and subsequently analyzed over a wavenumber range of 400–4000 cm^−1^. **Stability test** – The stability of Casein-PE@TNF-Ab in simulated biological fluids was evaluated by incubating the formulations in simulated gastric fluid (SGF) and simulated intestinal fluid (SIF) under physiological conditions (100 rpm, 37 °C). SGF was prepared by dissolving 2 g/L NaCl and adjusting the pH to 1.4, with or without 3.2 g/L pepsin. SIF was prepared using 6.8 g/L KH_2_PO_4_ adjusted to pH 6.8, with or without 10 g/L pancreatin, following a previously reported method (Kim et al., 2020). At predetermined time points, samples were collected and analyzed for particle size and zeta potential to assess stability. Additionally, SDS-PAGE was performed to evaluate the structural stability of Casein-PE@TNF-Ab and free TNF-Ab following treatment with SGF (with or without pepsin) and SIF (with or without pancreatin). Each sample was mixed with Tris-Glycine SDS sample buffer and NuPAGE^™^ sample reducing agent, followed by heating at 80 °C for 10 min. Subsequently, 10 μL of each sample was loaded onto a 4%–20% gradient polyacrylamide gel and electrophoresed using a Mini Gel Tank system (Invitrogen) at a constant voltage of 225 V for 35 min. Residual activity (dialysis-based) study – Free TNF-Ab and Casein-PE@TNF-Ab were loaded into 300 kDa dialysis bags and incubated in 45 mL simulated gastric fluid (SGF) at 37 °C with shaking at 100 rpm. After 2 h incubation, the contents remaining inside the dialysis bags were collected. Residual TNF-Ab activity was evaluated using an LPS-stimulated RAW cell model by measuring the inhibition of TNF-α production. In parallel, separate dialysis bags were incubated in SGF for 2 h under the same conditions and then transferred to fresh simulated intestinal fluid (SIF), followed by further incubation for an additional 22 h or 46 h. The contents remaining inside the dialysis bags were subsequently collected and analyzed using the same assay.

### 2.4. Uptake of Casein-PE@TNF-Ab by RAW 264.7

To investigate the cellular uptake of Casein-PE@TNF-Ab by RAW 264.7 macrophages, OVA-647 was used as a fluorescent surrogate for TNF-Ab to prepare Casein-PE@OVA-647. RAW 264.7 cells were seeded at a density of 5×10^4^ cells per well in a 48-well culture plate (200 μL/well) and incubated in high-glucose DMEM supplemented with 10% fetal bovine serum (FBS) and 1% penicillin–streptomycin overnight. On the following day, cells were divided into two groups: the control group received phosphate-buffered saline (PBS), and the LPS-treated group was stimulated with 200 ng/mL lipopolysaccharide (LPS). On the third day, free OVA-647 or Casein-PE@OVA-647 (equivalent to 5 μg/mL OVA-647) was added to both groups and incubated for 2 h at 37 °C. After incubation, cells were washed twice with PBS, detached, and fixed using fixation buffer (BioLegend). The cellular uptake of OVA-647 was quantified by flow cytometry (Cytek Aurora) based on Alexa Fluor^™^ 647 fluorescence. To further examine the intracellular localization of the formulation, RAW 264.7 cells were incubated with free OVA-647 or Casein-PE@OVA-647 under the same conditions as described above. Following incubation, cells were gently washed twice with PBS and stained with Hoechst 33,342 (Thermo Fisher) for 15 min to label the nuclei. After staining, cells were washed again with PBS and resuspended in 200 μL PBS. Fluorescence images were captured using a BioTek Cytation 7 imaging system.

### 2.5. Cell viability

The cytotoxicity of Casein-PE@TNF-Ab was evaluated in RAW 264.7 and Caco-2 cells using the LDH Cytotoxicity Assay Kit (Thermo Fisher Scientific). Cells were seeded at a density of 5 × 10^4^ cells per well in 100 μL of RPMI medium supplemented with 10% fetal bovine serum (FBS) and 1% penicillin–streptomycin in a 96-well plate and incubated for 2 h to allow adherence. Subsequently, 100 μL of medium containing various concentrations of Casein-PE@TNF-Ab was added to each well, resulting in a final volume of 200 μL per well. After 24 h of incubation at 37 °C, cell culture supernatants were collected, and LDH release was measured according to the manufacturer’s instructions to assess cell viability.

### 2.6. In vitro bioactivity assay of TNF-Ab

The bioactivity of Casein-PE@TNF-Ab was first evaluated by its ability to neutralize a known concentration of TNF-α, as measured by enzyme-linked immunosorbent assay (ELISA). Briefly, Casein-PE-TNF-Ab and free TNF-Ab were incubated with 125 pg/mL of recombinant TNF-α at 37 °C for 2 h to allow antigen–antibody binding. TNF-α bound to anti–TNF-α antibodies formed immune complexes and could not bind to the ELISA plate pre-coated with capture antibodies, thus being removed during the washing steps. The remaining unbound TNF-α in the supernatant, representing non-neutralized TNF-α, was quantified using a standard ELISA protocol. The TNF-α inhibition rate was calculated using the following formula:

$$TNF-\alpha \text{Inhibition}(\%)=\frac{125-\text{mesuredTNF}-\alpha }{125}\times 100$$

To further assess the ability of Casein-PE@TNF-Ab to neutralize endogenously produced TNF-α, RAW 264.7 macrophages were seeded at a density of 5 × 10^4^ cells per well in 100 μL of complete medium in 96-well plates. Cells were divided into three groups: LPS control group, Free TNF-Ab treatment group, and Casein-PE@TNF-Ab treatment group. After overnight incubation, all groups were stimulated with 200 ng/mL LPS. Simultaneously, the treatment groups received Casein-PE@TNF-Ab or free TNF-Ab at final concentrations of 10 or 20 μg/mL (based on TNF-Ab content), while the LPS control group received PBS. On day 3, supernatants were collected, and TNF-α levels were measured by ELISA to evaluate the neutralizing capacity of each formulation.

To evaluate whether Casein-PE protects the bioactivity of TNF-Ab under simulated gastrointestinal conditions, Casein-PE@TNF-Ab and free TNF-Ab were first incubated in SGF or SIF at 37 °C with shaking (100 rpm) for 2 h, followed by the same TNF-α neutralization assay.

### 2.7. In vivo distribution study

To assess the in vivo biodistribution of Casein-PE, the fluorescent model antigen OVA-Cy7 was synthesized using a previously reported method (Schwinghamer et al., 2024). Briefly, OVA was conjugated with Sulfo-Cyanine7 NHS ester in PBS (pH 8.0) by incubating the mixture overnight at room temperature in the dark. Unreacted dye and excess salts were removed using Zeba desalting columns (7 kDa MWCO; Thermo Fisher Scientific). To evaluate the biodistribution of Casein-PE in the model of colitis, acute colitis was induced in mice by administering 3.5% (w/v) DSS in drinking water for six consecutive days, followed by normal drinking water on day 7. On day 7, mice were administered either free OVA-Cy7 or Casein-PE@OVA-Cy7 by oral gavage (30 μg per mouse). At 4, 12, and 24 h after administration, mice were euthanized and the entire gastrointestinal tract was collected. Fluorescence imaging was performed using the Carestream In-Vivo MS FX PRO imaging system (Bruker) with an excitation wavelength of 750 nm and emission at 835 nm. X-ray images were acquired at 200 units, and overlay images were generated. Fluorescence intensity in the colon was quantified using Bruker’s analysis software. To further examine the tissue-level distribution of Cy7 fluorescence within the colon, a segment of the distal colon was collected immediately after sacrifice and fixed in 10% neutral buffered formalin for 1 h. The tissue was then rinsed three times with PBS and cryoprotected in 30% sucrose at 4 °C for 4 h. The samples were embedded in OCT compound, and 10-μm cryosections were prepared. Sections were mounted using ProLong^™^ Diamond Antifade Mountant with DAPI and imaged using a BioTek Cytation 7 plate reader to visualize Cy7 fluorescence.

### 2.8. In vivo treatment

#### 2.8.1. Animals

Female C57BL/6 immunocompetent mice (8–10 weeks old) were purchased from Charles River Laboratories (Wilmington, MA, USA) and housed in facilities managed by the Research Animal Resources Center at the University of Kansas. All animal experiments were performed in accordance with protocols approved by the University of Kansas Institutional Animal Care and Use Committee (IACUC, protocol number AUS 281–01).

#### 2.8.2. Induction of DSS-induced colitis and experimental design

Following a one-week acclimation period, mice were randomly divided into five groups (n = 5 per group): (i) PBS control (oral PBS), (ii) DSS group (3.5% DSS), (iii) Free TNF-Ab oral group (3.5% DSS + oral TNF-Ab, 5 mg/kg), (iv) Casein-PE@TNF-Ab oral group (3.5% DSS + oral TNF-Ab, 5 mg/kg), and (v) Casein-PE@TNF-Ab subcutaneous group (3.5% DSS + subcutaneous TNF-Ab, 5 mg/kg). Colitis was induced by administering 3.5% DSS in drinking water from day 1 to day 6 in all groups except the PBS control, which received regular water. From day 7 to day 10, all mice were switched to regular water. Drug treatments were administered once daily from day 4 to day 10. Body weight was recorded daily throughout the experiment. On day 10, mice were euthanized and colons were collected to measure colon length.

#### 2.8.3. Histopathology analysis and assessment of cytokines

Distal colon tissues were collected and processed by fixation in 10% formalin, followed by paraffin embedding and sectioning. Tissue sections were stained with hematoxylin and eosin (H&E) for histological evaluation, and images were acquired using the BioTek Citations 7 imaging system. For cytokine analysis, colonic tissues were homogenized to obtain tissue lysates. Briefly, colon segments were minced into small pieces and suspended in phosphate-buffered saline (PBS) at a concentration of 30 mg/mL. Tissue homogenization was performed using a BeadBug 6 microtube homogenizer (Benchmark, Tempe, AZ) for 15 min at 4350 rpm. The homogenates were then centrifuged at 12,000 rpm for 5 min at 4 °C, and the supernatants were collected for further analysis. Cytokine levels, including TNF-α and interleukin-1β (IL-1β), were quantified using ELISA kits (BioLegend) according to the manufacturer’s instructions. Myeloperoxidase (MPO) levels were determined using the Invitrogen^™^ Mouse Myeloperoxidase ELISA Kit.

#### 2.8.4. Flow cytometry analysis

To assess the modulatory effect of orally administered Casein-PE@TNF-Ab on colonic neutrophil infiltration, flow cytometry was used to analyze neutrophil populations in colon tissue. Colon tissues were harvested and cut into small fragments, followed by incubation in mucosal removal buffer containing HBSS, 5% fetal bovine serum (FBS), 1 mM dithiothreitol (DTT), and 5 mM EDTA at 37 °C for 45 min to remove epithelial cells. After rinsing with PBS, the tissues were transferred to digestion buffer composed of HBSS with 5% FBS, 0.8 mg/mL Collagenase A, and 10 μg/mL DNase I, and incubated at 37 °C for 1 h. The digested tissue was further dissociated mechanically using the gentleMACS^™^ Octo Dissociator with Heaters (Miltenyi Biotec). The resulting cell suspensions were passed through 70-μm cell strainers to remove debris and then centrifuged. Pelleted cells were resuspended in PBS containing 1 mM EDTA and 1% FBS and incubated at room temperature for 15 min, followed by a second centrifugation. Cells were then resuspended in cell staining buffer (biolegend) and stained with fluorophore-conjugated anti-mouse antibodies: CD45-PerCP, CD11b-Fluor^®^ 700, and Ly-6G-PE. Flow cytometry was performed using the Cytek Aurora system, and data were analyzed with FlowJo software (version 10.8.1).

#### 2.8.5. Statistical analysis

Statistical analyses were performed using GraphPad Prism 10.0.0. One-way analysis of variance (ANOVA) was used for comparisons among multiple groups, while Student’s *t*-test was applied when comparing two groups.

## 3. Results

### 3.1. Fabrication and characterization of Casein-PE@TNF-Ab

**Fig. 1.**
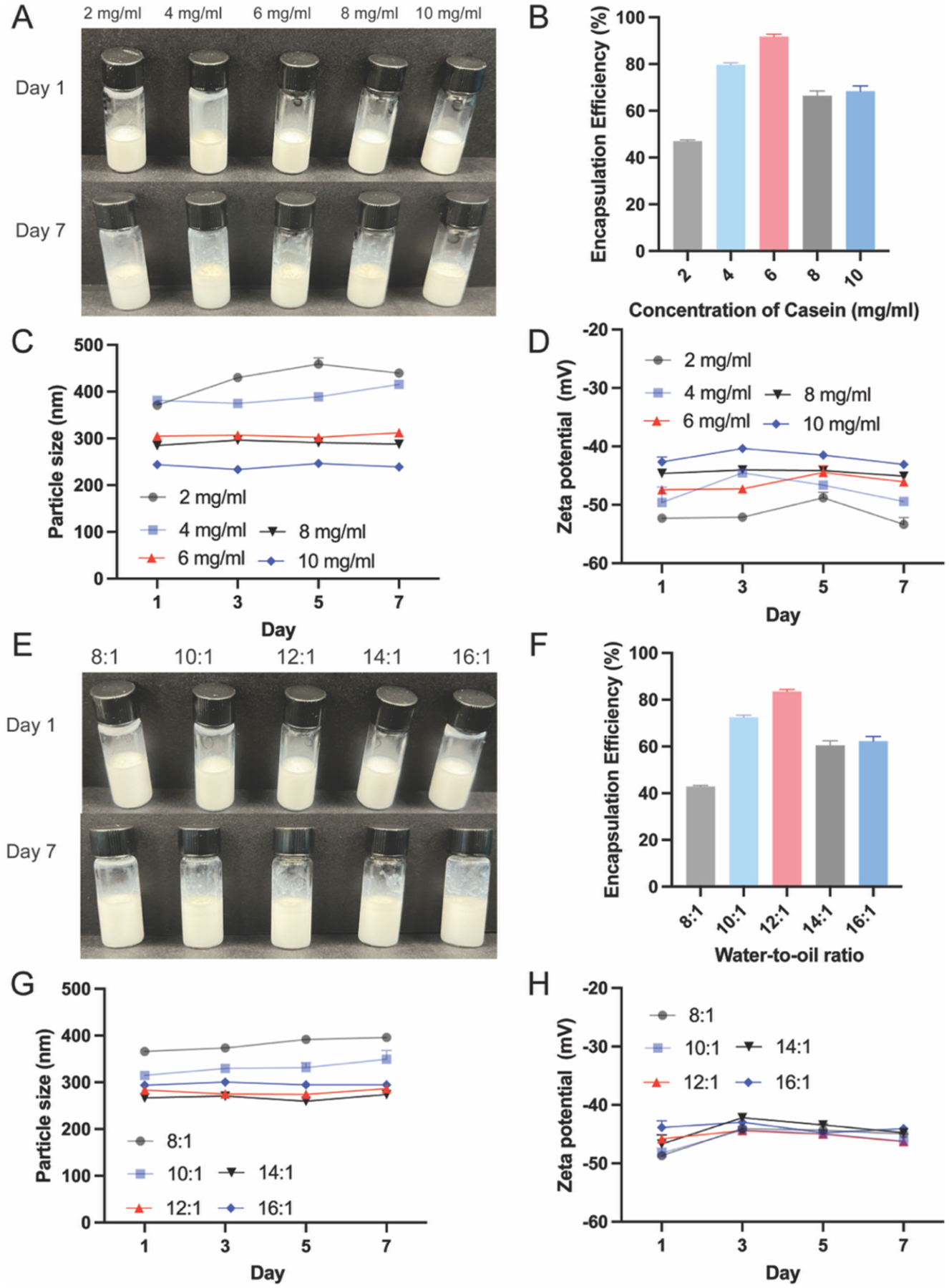
Optimization of Casein-PE@TNF-Ab. (A–D) Effects of varying casein concentration on the appearance, encapsulation efficiency, particle size, and zeta potential of Casein-PE@TNF-Ab. (E–H) Optimization of oil-to-water ratio to fabricate Casein-PE@TNF-Ab. Effects of varying oil-to-water ratios on the appearance, encapsulation efficiency, particle size, and zeta potential of Casein-PE@TNF-Ab. n = 3, All data are represented as mean ± SD.

To optimize the Casein-PE formulation, Pickering emulsions were prepared with varying oil-to-water ratios and casein concentrations. The formulations were evaluated for visual stability, antibody loading efficiency, particle size, and zeta potential. In the casein concentration screening, emulsions prepared with 2 and 4 mg/mL casein exhibited visible phase separation within 7 days (Fig. 1A), while those prepared with 6, 8, and 10 mg/mL remained visually stable, retaining a uniform milky-white appearance without signs of stratification or aggregation. These results suggest that at low casein concentrations, the amount of stabilizer is insufficient to maintain interfacial stability, leading to emulsion destabilization. Consistently, the 2 and 4 mg/mL groups also showed lower antibody encapsulation efficiencies on day 7 (Fig. 1B). Among the stable groups, the formulation containing 6 mg/mL casein exhibited the highest encapsulation efficiency, indicating that casein concentration must be appropriately balanced—insufficient concentrations result in instability, while excessive concentrations may lead to saturation of the interfacial area, thereby limiting antibody adsorption and reducing encapsulation efficiency. Particle size and zeta potential measurements showed that emulsions containing 6–10 mg/mL casein remained colloidally stable over 7 days, with mean particle sizes around 300 nm and zeta potentials near − 45 mV. Specifically, the 6 mg/mL formulation exhibited a particle size of 304.8 ± 5.6 nm and a zeta potential of − 47.41 ± 0.79 mV on day 7 (Fig. 1C–D). Based on these results, 6 mg/mL casein was selected for further development.

Subsequently, emulsions were prepared using different water-to-oil ratios ranging from 8:1 to 16:1 to assess their effect on formulation performance. After 7 days of storage at 4 °C, all emulsions appeared as homogeneous milky-white dispersions (Fig. 1E). Among these, the formulation with a 12:1 water-to-oil ratio achieved the highest antibody encapsulation efficiency—1.97-fold higher than 8:1, 1.15-fold higher than 10:1, 1.38-fold higher than 14:1, and 1.33-fold higher than 16:1 (Fig. 1F). Notably, the 8:1 group exhibited the lowest encapsulation efficiency (~40%), which may be attributed to excessive oil content increasing interfacial hydrophobicity, thereby reducing adsorption of hydrophilic antibodies. Additionally, this group showed a particle size increase from 366.1 nm on day 1 to 391.8 nm on day 7 (Fig. 1G), suggesting mild aggregation, which may further explain the reduced encapsulation efficiency. In contrast, emulsions prepared with other ratios maintained stable particle size and zeta potential throughout the storage period (Fig. 1G–H), indicating satisfactory colloidal stability. Based on these findings, the optimized Casein-PE-TNF-Ab formulation was prepared using a 12:1 water-to-oil ratio and 6 mg/mL casein, which resulted in a mean particle size of 283.4 ± 4.0 nm, a zeta potential of − 45.8 ± 0.6 mV, and an encapsulation efficiency of 83.5 ± 0.9%.

To verify the successful encapsulation of TNF-Ab within the formulation, Fourier-transform infrared (FT-IR) spectroscopy was performed. Supplementary Fig. 1 displays the infrared spectra of free casein, free TNF-Ab, and Casein-PE@TNF-Ab. The spectrum of casein exhibited characteristic absorption bands at 3300 cm^−1^ (N–H stretching), 1630 cm^−1^ (C=O stretching, Amide I), 1511 cm^−1^ (N–H bending and C–N stretching, Amide II), and 1078 cm^−1^ (P=O stretching), which are consistent with typical protein signatures. Similarly, the TNF-Ab spectrum showed N–H stretching at 3300 cm^−1^, C=O stretching at 1650 cm^−1^, C–O/C–N stretching at 1078 cm^−1^, and a distinct absorption at 526 cm^−1^ corresponding to disulfide (S–S) bond vibrations. The FT-IR spectrum of Casein-PE@TNF-Ab displayed characteristic peaks matching those of both free casein and TNF-Ab, confirming the successful incorporation of TNF-Ab into the formulation.

### 3.2. The stability and release of Casein-PE@TNF-Ab

**Fig. 2.**
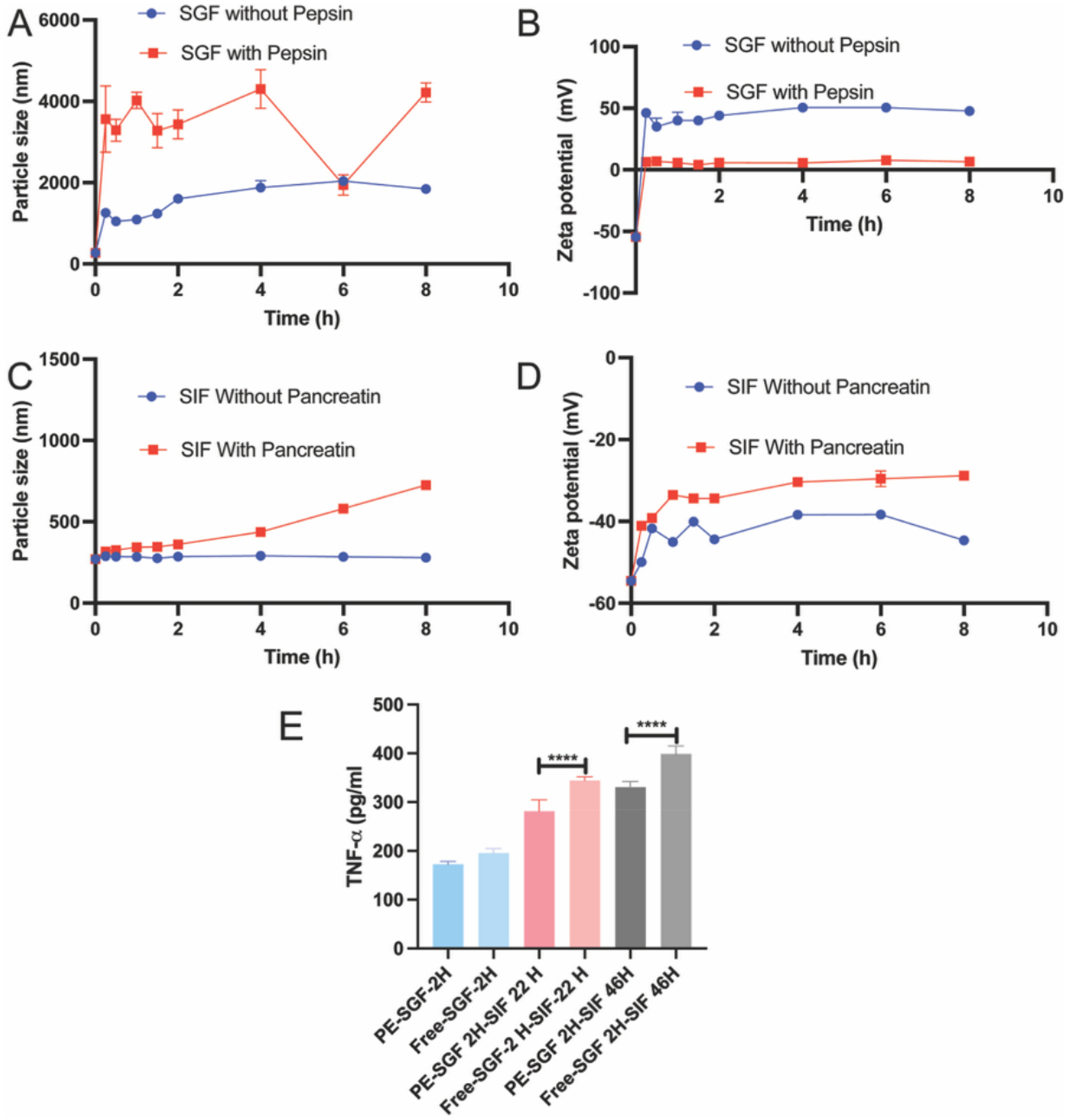
Stability and of Casein-PE@TNF-Ab. (A-D) Stability assessment of Casein-PE@TNF-Ab in simulated gastric fluid (SGF) and simulated intestinal fluid (SIF) by measuring particle size and zeta potential. n = 3, All data are represented as mean ± SD. (E) Residual TNF-Ab activity after incubation in SGF (2 h) and SIF (22 or 46 h). Samples from dialysis bags were analyzed using an LPS-stimulated RAW cell model. ****P < 0.0001. n = 3, data are presented as mean ± SEM. Data were analyzed by one-way ANOVA.

To evaluate the gastrointestinal stability of the Casein-PE@TNF-Ab formulation, the optimized emulsion was incubated in SGF and SIF, with or without digestive enzymes. In SGF without pepsin (Fig. 2A), the particle size increased from 270 nm at 15 min to over 1000 nm, and and further reaching approximately 1800 nm after 8 h. This increase may be attributed to the formation of a clot-like network by casein under acidic conditions, leading to particle aggregation. The zeta potential (Fig. 2B) shifted from − 54.51 mV to + 56.17 mV, likely due to the adsorption of H^+^ ions onto the emulsion surface, resulting in charge reversal. Over the following 8 h, the zeta potential stabilized at approximately + 45 mV, and the absolute value above 40 mV suggests that the formulation remained relatively stable despite the acidic environment. In SGF with pepsin (Fig. 2A), the particle size further increased to approximately 3000 nm, possibly due to pepsin-mediated hydrolysis of casein, which disrupted the interfacial structure and promoted droplet aggregation. The zeta potential decreased markedly from − 54.51 mV to around 5 mV, indicating a loss of surface charge and electrostatic repulsion, which could further compromise colloidal stability. In SIF without pancreatin (Fig. 2C), the particle size remained stable at approximately 280 nm throughout the 8-hour incubation, and the zeta potential (Fig. 2D) was consistently maintained around − 40 mV, indicating good physical stability under neutral conditions. In SIF with pancreatin, the particle size was maintained around 300 nm during the first 2 h, increased to 436 nm by 4 h, and reached 726 nm after 8 h. Meanwhile, the zeta potential remained relatively stable around − 30 mV over the entire incubation period. These results indicate that the Casein-PE@TNF-Ab formulation demonstrates good colloidal stability in both enzyme-free and enzyme-containing intestinal environments, supporting its potential as an oral delivery system.

To determine whether the increase in particle size after incubation with SGF was associated with casein aggregation under acidic conditions, blank casein was incubated with SGF or SIF and subsequently characterized by microscopy, particle size analysis, and optical density (OD) measurements. As shown in Supplementary 2 Fig. A, incubation in SGF resulted in the formation of a dense aggregated network structure, indicative of a casein clot-like morphology under acidic conditions. In contrast, no obvious structural aggregation was observed in samples incubated with SIF. Consistent with the microscopic observations, particle size analysis (Supplementary Fig. B) showed that the average particle size increased markedly from approximately 240 nm to 1477 nm after incubation with SGF, whereas the particle size remained around 240 nm following incubation with SIF. Furthermore, OD measurements at 400 nm (Supplementary Fig. C–D) showed that the OD values of blank SGF, blank SIF, and SIF-incubated casein were approximately 0.05, while incubation of casein with SGF resulted in a significantly higher OD value (~0.2, ****P < 0.0001). These results indicate that acidic conditions promote the formation of a casein clot-like aggregated network, which likely contributes to the observed increase in particle size.

SDS-PAGE analysis was performed to evaluate the structural stability of Casein-PE@TNF-Ab. As shown in Supplementary Fig. 3, the free TNF-Ab (line a) displayed two distinct bands corresponding to the heavy chain (~50 kDa) and light chain (~25 kDa). The blank Casein-PE (line F) exhibited two bands around 31–36 kDa, characteristic of casein proteins. The Casein-PE@TNF-Ab formulation (line A) showed bands corresponding to both TNF-Ab and casein, indicating that the antibody’s structural integrity was preserved during the formulation process. After treatment with SGF, both with and without pepsin, the free TNF-Ab (lines B–C) and Casein-PE@TNF-Ab (lines B–C) exhibited similar degradation patterns. In SGF without pepsin, the 50 kDa heavy chain band appeared slightly blurred and the light chain band was faint, suggesting minor degradation. In contrast, in SGF with pepsin, both heavy and light chains were completely degraded, indicating that pepsin has a strong proteolytic effect on TNF-Ab. Following incubation in simulated intestinal fluid (SIF) without pancreatin, both free TNF-Ab (line D) and Casein-PE@TNF-Ab (line D) retained their original heavy and light chain bands, suggesting preserved structural integrity. Notably, after treatment with SIF containing pancreatin, the bands of Casein-PE@TNF-Ab were more intense than those of free TNF-Ab, indicating that the Casein-PE formulation provides a protective effect against pancreatin-mediated degradation.

As shown in the Fig. 2E, after 2 h incubation in SGF, the TNF-Ab retained in the dialysis bag in the Casein-PE@TNF-Ab group exhibited higher TNF-α neutralization activity compared to free TNF-Ab. Following sequential incubation (2 h in SGF followed by 22 h in SIF), the residual TNF-Ab activity in the Casein-PE group remained significantly higher than that of the free TNF-Ab group (*P* < 0.0001), with TNF-α levels reduced to approximately 81% of those observed in the free antibody group. A similar trend was observed after 46 h incubation in SIF (*P* < 0.0001). These results indicate that Casein-PE enhances the stability of TNF-Ab under SGF conditions. In addition, the higher residual activity observed within the dialysis bags suggests a slower release behavior in SIF.

To evaluate long-term stability, Casein-PE was stored at 4 °C for 3 months and assessed for visual appearance, particle size, PDI, and zeta potential, as well as bioactivity of encapsulated antibody. After storage, the emulsion remained uniformly milky without visible creaming or aggregation (Supplementary Fig. 4 A). The particle size and PDI were 327.8 nm and 0.264, respectively, indicating a narrow size distribution. The zeta potential was − 40.81 mV (Supplementary Fig. 4B), consistent with electrostatic stabilization.

### 3.3. Cellular uptake of Casein-PE@TNF-Ab

**Fig. 3.**
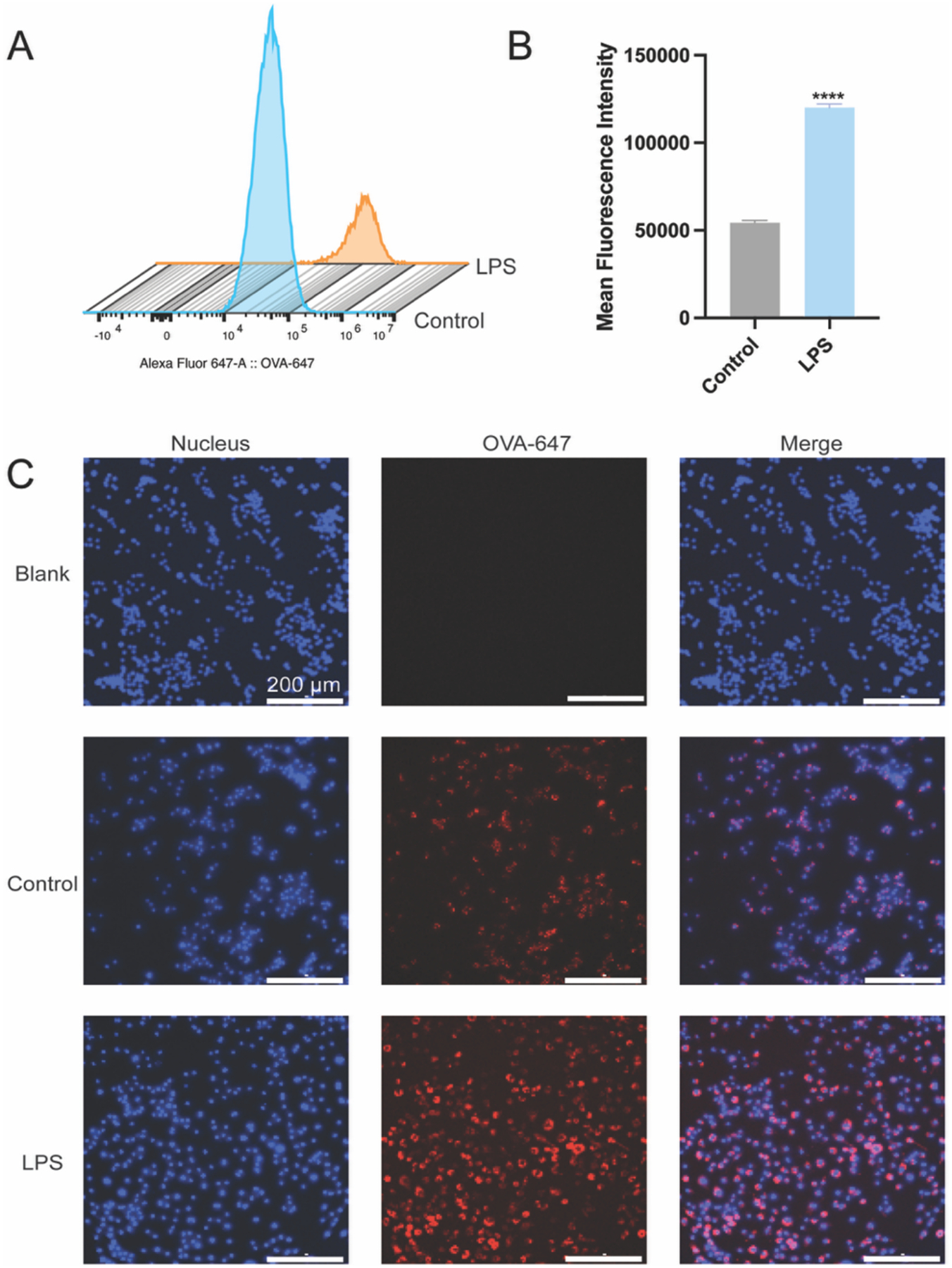
Uptake of Casein-PE@OVA-647 by RAW cells. (A) Histogram of OVA-647 signal intensities from RAW cells (B) Quantitative analysis of cellular uptake by flow cytometry (mean ± SD, n = 4), Student’s *t*-test ****P < 0.0001 (C) Fluorescence microscopy images of RAW cells incubated with Casein-PE@OVA-647 for 2 h under LPS-stimulated and unstimulated conditions. Scale bar: 200 μm.

To assess whether Casein-PE encapsulation of TNF-Ab can enhance antigen uptake by LPS-stimulated RAW264.7 cells, a cellular uptake study was conducted using fluorophore-labeled ovalbumin (OVA-647). As shown in Supplementary Fig. 5 and Table 1, Casein-PE@OVA-647 exhibited a particle size of 326 nm (PDI = 0.215) and a zeta potential of − 45.7 mV, comparable to Casein-PE@TNF-Ab, indicating that fluorescent labeling and the use of OVA-647 as a surrogate cargo did not significantly affect the physicochemical properties of the formulation. As shown in Fig. 3A–B, LPS stimulation significantly enhanced the uptake of OVA compared to the untreated control group, with fluorescence intensity reaching 2.2 times that of the control. Consistently, fluorescence microscopy further confirmed this finding, showing markedly higher OVA (red) fluorescence in LPS-stimulated RAW cells than in the control group (Fig. 3C). Collectively, these results indicate that Casein-PE@OVA-647 promotes enhanced antigen uptake by LPS-stimulated macrophages.

### 3.4. TNF-α neutralization activity and cellular toxicity of Casein-PE@TNF-Ab

**Fig. 4.**
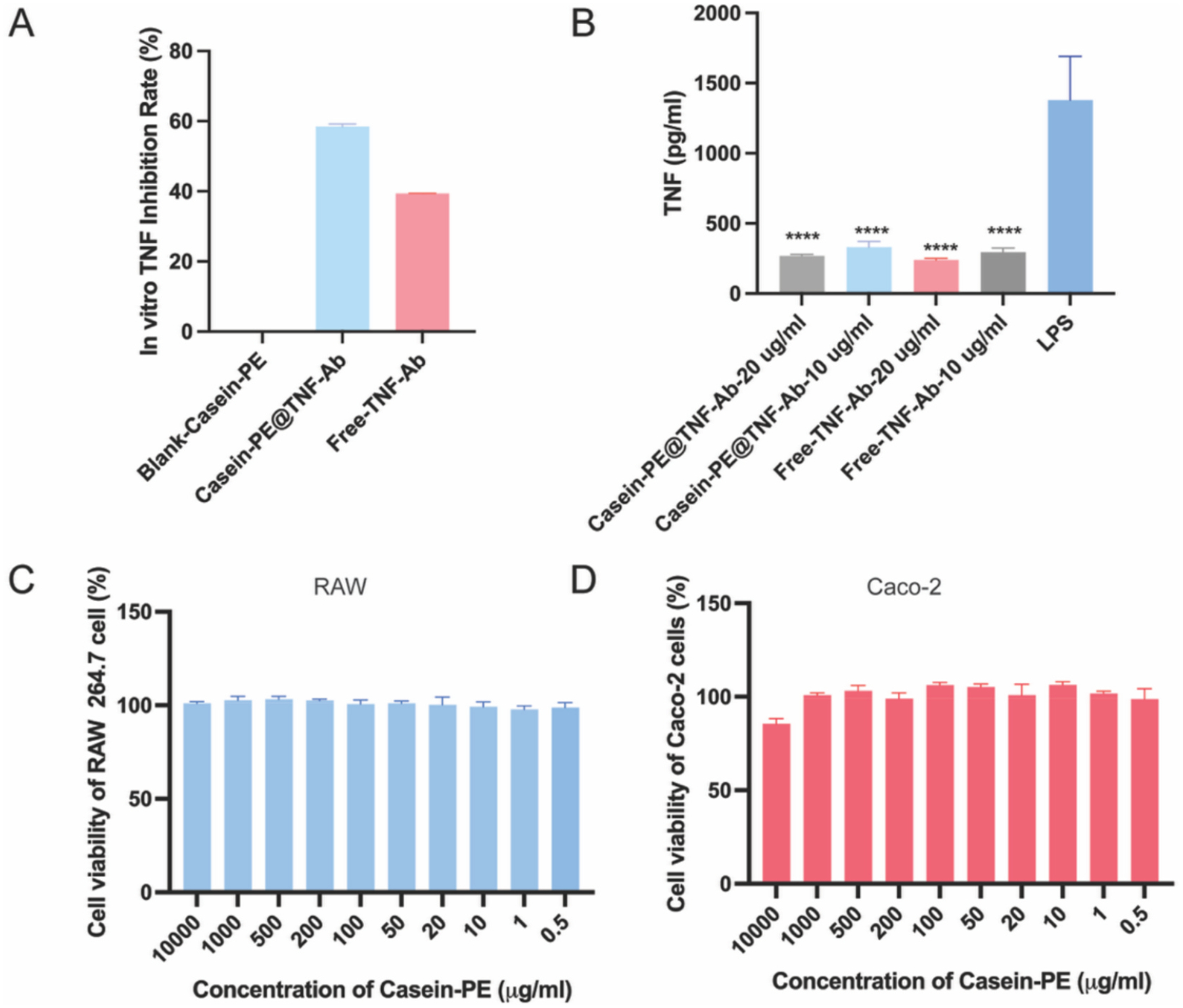
(A) In vitro TNF-α inhibition by Casein-PE@TNF-Ab. (B) Neutralization of TNF-α by Casein-PE@TNF-Ab in LPS-stimulated RAW 264.7 cells. (C–D) Cell viability of RAW 264.7 and Caco-2 cells after 24-hour treatment with Casein-PE@TNF-Ab. ****P < 0.0001. n = 4, All data are represented as mean ± SD. Data were analyzed by one-way ANOVA.

To assess whether the TNF-α neutralization activity of TNF-Ab is retained after encapsulation into the Casein-PE system, we evaluated its ability to neutralize both exogenously added TNF-α and TNF-α secreted by LPS-stimulated RAW264.7 cells. As shown in Fig. 4A, blank Casein-PE showed no TNF-α neutralizing activity. In contrast, Casein-PE@TNF-Ab achieved a TNF-α inhibition rate of 58.3%, compared with 39.3% observed for free TNF-Ab, indicating that encapsulation into Casein-PE preserves and may even enhance the in vitro neutralization capacity of TNF-Ab. Consistently, treatment with Casein-PE@TNF-Ab (10 μg/mL and 20 μg/mL), as well as free TNF-Ab, significantly reduced TNF-α levels in LPS-stimulated RAW264.7 cells compared with the LPS-only group (*P* < 0.001), further confirming effective TNF-α neutralization activity.

To further evaluate whether Casein-PE protects the bioactivity of TNF-Ab under simulated gastrointestinal conditions, Casein-PE@TNF-Ab and free TNF-Ab were incubated in SGF or SIF for 2 h prior to the neutralization assays. As shown in Supplementary Fig. 6 A, after incubation in SGF for 2 h, Casein-PE@TNF-Ab retained a TNF-α inhibition rate of 50.5%. In contrast, free TNF-Ab exhibited a significantly reduced inhibition rate (40.2%, *P* < 0.0001), suggesting partial loss of neutralizing activity under acidic gastric conditions. After incubation in SIF for 2 h, Casein-PE@TNF-Ab and free TNF-Ab displayed comparable TNF-α inhibition rates.

Consistent trends were observed in the cellular assay. Both Casein-PE@TNF-Ab and free TNF-Ab incubated in SGF or SIF significantly reduced TNF-α levels in LPS-stimulated RAW264.7 cells compared with the LPS group (*P* < 0.0001). Notably, TNF-α levels in the group treated with SGF-incubated free TNF-Ab were significantly higher than those in the other three treatment groups (*P* < 0.001) and were approximately threefold higher than those observed with Casein-PE@TNF-Ab, indicating partial loss of neutralizing activity of free TNF-Ab after SGF exposure.

To evaluate storage stability, Casein-PE was stored at 4 °C for 3 months and subsequently loaded with TNF-Ab. In the LPS-stimulated RAW264.7 assay, treatment at 10–20 μg/mL significantly reduced TNF-α levels compared with the LPS group (*P* < 0.001; Supplementary Fig. 4C), indicating that prolonged refrigerated storage does not compromise the loading capacity of the Casein-PE carrier or the TNF-α–neutralizing activity of the resulting formulation.

Additionally, the cytocompatibility of Casein-PE was evaluated in RAW264.7 and Caco-2 cells (Fig. 4C–D). Both cell types maintained nearly 100% viability after overnight incubation with Casein-PE across a wide concentration range (0.5–1000 μg/mL), indicating excellent cytocompatibility of the formulation.

### 3.5. In vivo biodistribution studies

To evaluate whether Casein-PE enhances the colonic retention of the antibody in DSS-induced colitis, in vivo fluorescence imaging was performed using OVA-Cy7 as a fluorescent surrogate for TNF-Ab. Casein-PE@OVA-Cy7 exhibited a particle size of 324 nm (PDI = 0.223) and a zeta potential of − 52.0 mV, comparable to those of Casein-PE@TNF-Ab, confirming that OVA-Cy7 can serve as a suitable surrogate cargo for TNF-Ab in the in vivo imaging study.

**Fig. 5.**
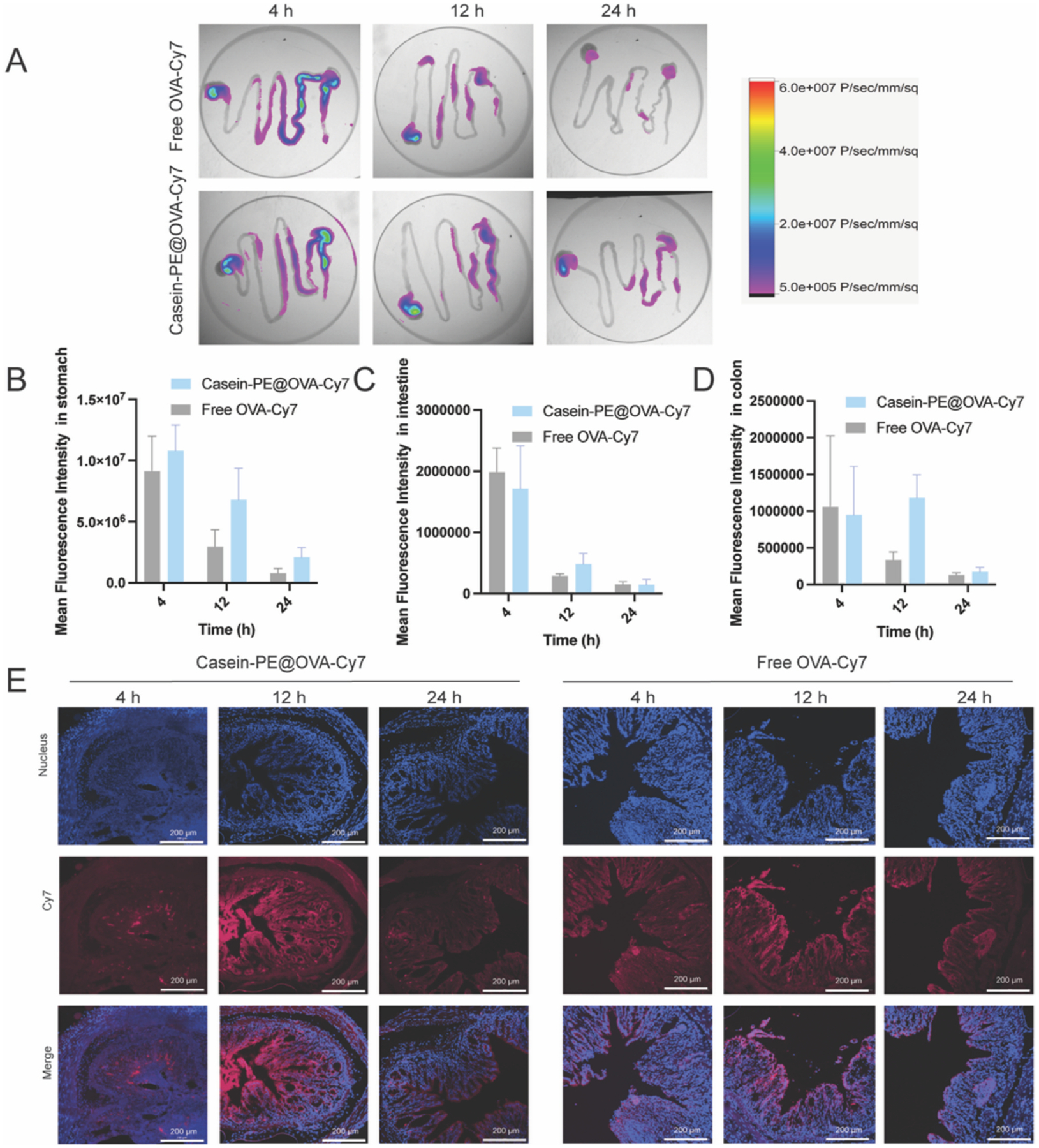
(A) Representative ex vivo fluorescence images of colons from DSS-induced colitis mice at 4, 12, and 24 h after oral gavage of Free OVA-Cy7 or Casein-PE@OVA-Cy7. (B–D) Quantification of fluorescence intensity in the stomach (B), intestine (C), and colon (D). n = 3; data are presented as mean ± SEM. (E) Representative colon sections at 4, 12, and 24 h after oral gavage of Free OVA-Cy7 or Casein-PE@OVA-Cy7; scale bar, 200 μm.

As shown in Fig. 5A–B, at 4 h after oral gavage the overall fluorescence signals of free OVA-Cy7 and Casein-PE@OVA-Cy7 were comparable. At later time points (12 and 24 h), Casein-PE@OVA-Cy7 exhibited stronger fluorescence signals throughout the gastrointestinal tract than free OVA-Cy7, suggesting protection of the antigen from gastric degradation and a sustained release profile. Notably, relatively strong fluorescence signals were still observed in the stomach at 24 h, indicating that part of the formulation remained in the upper gastrointestinal tract and served as a reservoir for gradual downstream release during gastrointestinal transit. This phenomenon may be associated with the ability of casein to form a clot-like aggregated network under acidic conditions (Supplementary Fig.2), which can temporarily retain the formulation in the stomach and enable sustained release during gastrointestinal transit.

Consistently, fluorescence signals in the colon (Fig. 5A, D) increased over time in the Casein-PE@OVA-Cy7 group. At 12 h, the colonic fluorescence intensity was 3.51-fold higher than that of the free OVA-Cy7 group and remained 1.34-fold higher at 24 h, indicating enhanced delivery and retention of the formulation in the colon. Cryosectioning of colon tissues (Fig. 5E) further confirmed stronger tissue-associated fluorescence in mice treated with Casein-PE@OVA-Cy7 at later time points. Together, these results suggest that Casein-PE prolongs gastrointestinal residence and facilitates sustained downstream transport, ultimately enhancing colonic exposure and retention of the encapsulated cargo in DSS-induced colitis mice.

### 3.6. Therapeutic effect of Casein-PE@TNF-Ab on DSS-Induced colitis

**Fig. 6.**
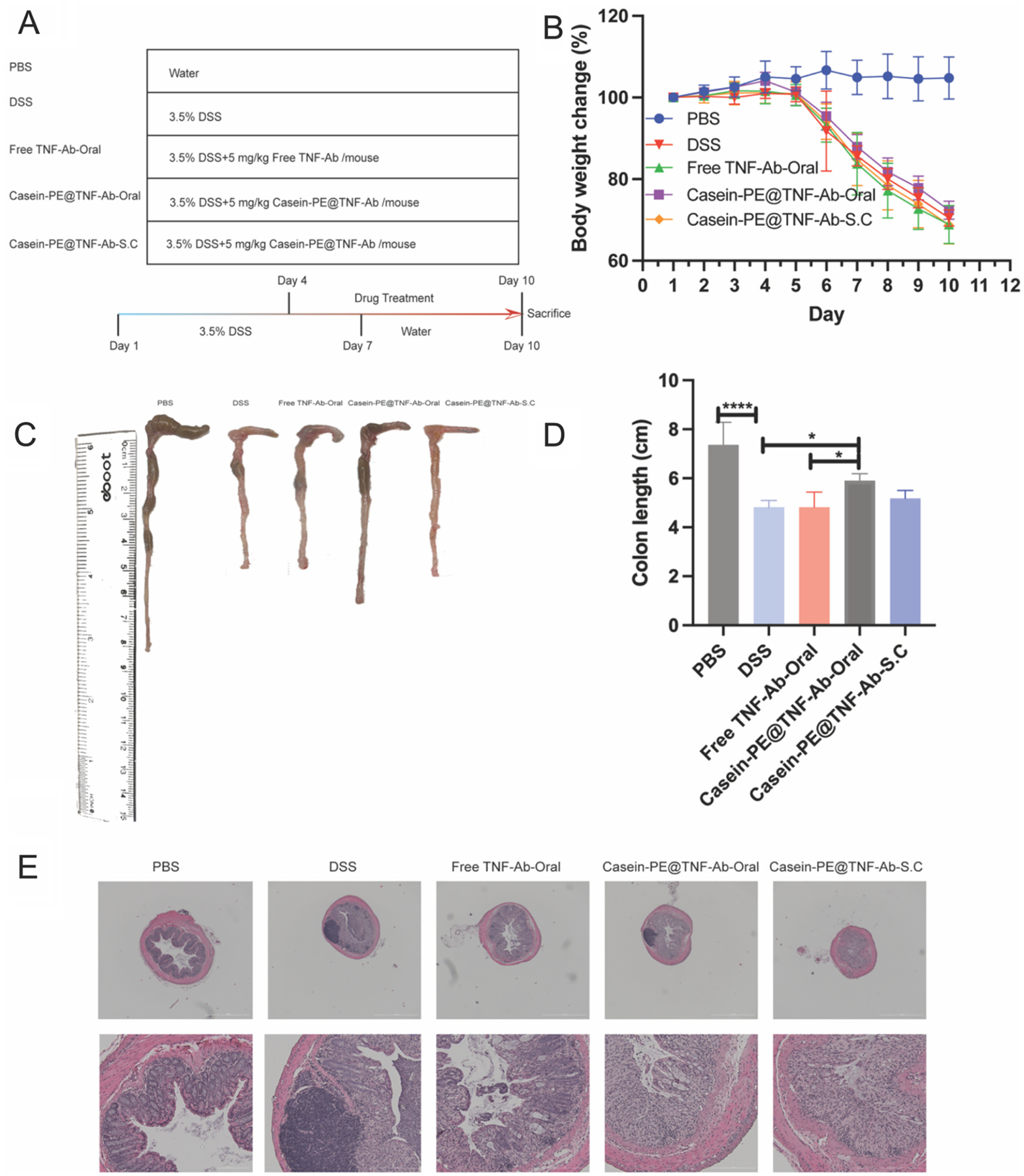
(A) Schematic diagram of DSS-induced colitis model establishment and treatment schedule, including dosage and group allocation. (B) Changes in body weight (% of initial body weight). (C–D) Representative images of colonic morphology and corresponding statistical analysis of colon length. (E) Representative H&E-stained sections of the proximal colon. *P < 0.05, ****P < 0.0001. n = 5, data are presented as mean ± SEM. Data were analyzed by one-way ANOVA.

To evaluate the therapeutic efficacy of Casein-PE@TNF-Ab in a DSS-induced colitis mouse model, mice were randomly divided into five groups and administered either free TNF-Ab or Casein-PE@TNF-Ab, as illustrated in Fig. 6A. As shown in Fig. 6B, the body weight of mice in the DSS group began to decline noticeably on day 6 and had decreased by approximately 30% by day 10, confirming successful model establishment. However, none of the treatment groups showed significant improvement in preventing body weight loss compared to the DSS group. Colon length measurements (Fig. 6C–D) further supported successful model induction, as the DSS group exhibited significantly shortened colons compared to the PBS control group (*P* < 0.0001). Among the treatment groups, only the oral Casein-PE@TNF-Ab group showed a significant increase in colon length compared to the DSS group (*P* < 0.05), reaching 1.22-fold that of the DSS group. Notably, when comparing across treatment groups, the oral Casein-PE@TNF-Ab group exhibited the greatest restoration of colon length, with a statistically significant difference compared to the oral free TNF-Ab group (*P* < 0.05). Histological evaluation of distal colon sections by hematoxylin and eosin (H&E) staining (Fig. 6E) revealed severe inflammatory cell infiltration, extensive mucosal damage, partial goblet cell loss, and crypt disruption in the DSS group. In contrast, both the oral and subcutaneous Casein-PE@TNF-Ab groups showed relatively preserved colonic architecture and reduced inflammatory injury.

### 3.7. Casein-PE@TNF-Ab reduces the expression of inflammatory cytokines and MPO in the colon

**Fig. 7.**
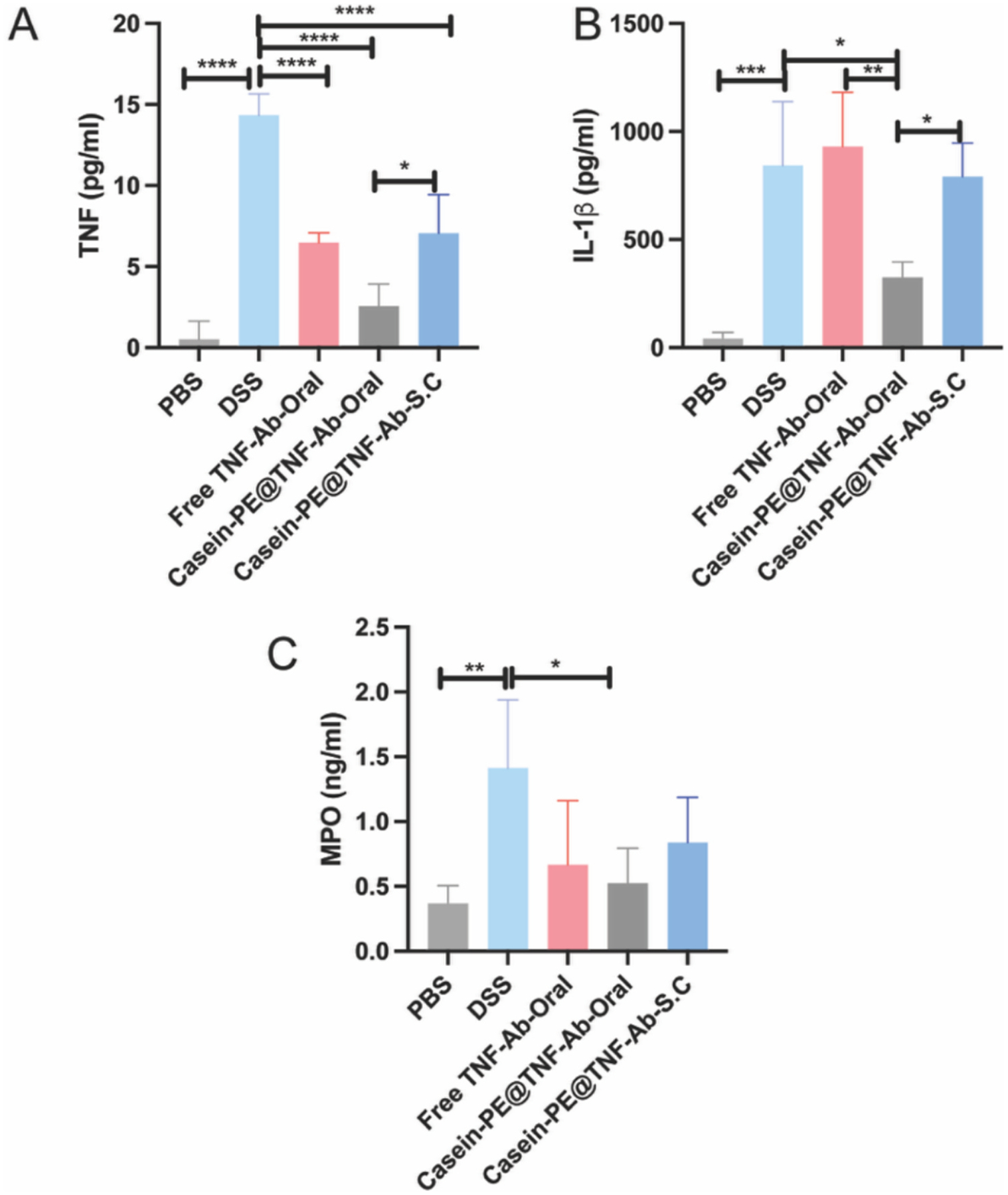
The expression of TNF-α, IL-1β, and MPO in colon tissue. *P < 0.05, **P < 0.01, ***P < 0.001, ****P < 0.0001. n = 5, data are presented as mean ± SEM. Data were analyzed by one-way ANOVA.

The therapeutic efficacy of Casein-PE@TNF-Ab against colonic inflammation was further evaluated by quantifying pro-inflammatory cytokines and myeloperoxidase (MPO) levels using ELISA kits. As shown in Fig. 7A, the DSS group exhibited a significant upregulation of TNF-α expression compared to the PBS group (*P* < 0.0001). Treatment with free TNF-Ab (oral), Casein-PE@TNF-Ab (subcutaneous), and Casein-PE@TNF-Ab (oral) significantly reduced TNF-α levels relative to the DSS group (*P* < 0.0001). The effect was most pronounced with oral Casein-PE@TNF-Ab, which reduced TNF-α by 82% compared with DSS. Moreover, TNF-α levels in the oral Casein-PE@TNF-Ab group were 60% lower than in the free TNF-Ab group (*P* < 0.05). A similar trend was observed for IL-1β expression (Fig. 7B). DSS-treated mice exhibited a significant increase in IL-1β compared to the PBS group (*P* < 0.001). Oral administration of Casein-PE@TNF-Ab reduced IL-1β levels by 61% relative to the DSS group, whereas oral free TNF-Ab produced no significant effect. Importantly, oral Casein-PE@TNF-Ab significantly reduced IL-1β levels compared with both the free TNF-Ab (oral, *P* < 0.01) and the Casein-PE@TNF-Ab (S.C, *P* < 0.05) groups, by 65% and 59%, respectively. In addition, MPO levels were significantly elevated in the DSS group compared to the PBS group (*P* < 0.01) (Fig. 7C). While all three treatment groups demonstrated a reduction in MPO expression, only the oral Casein-PE@TNF-Ab group showed a statistically significant decrease (*P* < 0.05), reducing MPO by 62% compared with the DSS group.

To further compare the therapeutic efficacy of oral Casein-PE@TNF-Ab with systemic TNF-Ab administration, an additional animal experiment was performed. As shown in Supplementary Fig. 7A, oral Casein-PE@TNF-Ab significantly reduced colonic TNF-α expression compared with the DSS group (*P* < 0.01), whereas intraperitoneal (i.p.) administration of free TNF-Ab resulted in a reduction that did not reach statistical significance. Notably, TNF-α levels in the oral Casein-PE@TNF-Ab group were approximately 65% lower than those in the i.p. free TNF-Ab group. A similar trend was observed for IFN-γ expression (Supplementary Fig. 7B). Both oral Casein-PE@TNF-Ab and i.p. free TNF-Ab significantly reduced IFN-γ levels compared with the DSS group (P < 0.001), with oral Casein-PE@TNF-Ab showing a greater reduction (78%) than i.p. free TNF-Ab (66%). In addition, both treatments decreased MPO levels relative to the DSS group (Supplementary Fig. 7C), although statistical significance was observed only in the oral Casein-PE@TNF-Ab group (P < 0.05). Furthermore, oral Casein-PE@TNF-Ab markedly reduced IL-1β expression by 76% compared with the DSS group (*P* < 0.0001), whereas i.p. free TNF-Ab reduced IL-1β by 46% (*P* < 0.01) (Supplementary Fig. 7D). In addition, both i.p. free TNF-Ab and oral Casein-PE@TNF-Ab reduced IL-6 expression compared with the DSS group, with a greater reduction observed in the oral Casein-PE@TNF-Ab group (Supplementary Fig. 7E). Together, these results demonstrate that oral delivery of TNF-Ab via the Casein-PE platform effectively suppresses multiple pro-inflammatory mediators and provides anti-inflammatory efficacy comparable to systemic administration.

### 3.8. Casein-PE@TNF-Ab reduces neutrophil infiltration in the colon

**Fig. 8.**
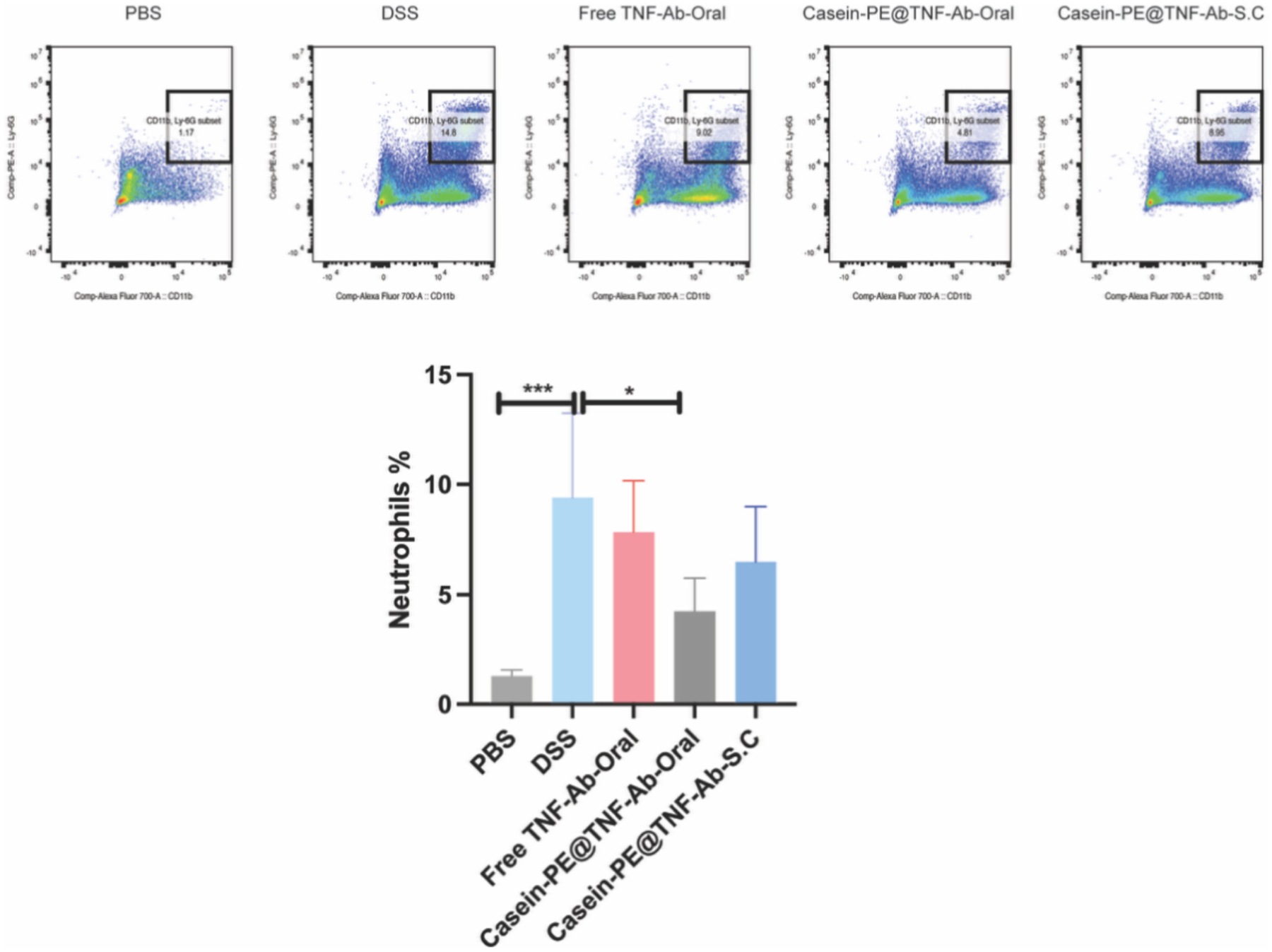
Representative flow cytometry dot plots and quantitative analysis of neutrophil populations in the colon. *P < 0.05, ***P < 0.001. n = 5, data are presented as mean ± SEM. Data were analyzed by one-way ANOVA.

TNF-α promotes neutrophil recruitment during inflammation, and TNF-Ab is expected to reduce this effect by neutralizing TNF-α (Little et al., 2006; Lukacs et al., 1995). We therefore evaluated whether TNF-Ab could decrease neutrophil infiltration, and whether encapsulation within Casein-PE (Casein-PE@TNF-Ab) could further enhance this effect. Colon neutrophil results (Fig. 8) showed that the DSS group exhibited a significant increase in neutrophil population compared to the PBS group (*P* < 0.001). While all treatment groups reduced neutrophil levels relative to the DSS group, only the Casein-PE@TNF-Ab group demonstrated a statistically significant reduction (*P* < 0.05), reducing the levels by 46%, 55%, and 66% compared with the DSS group, the free TNF-Ab group, and the Casein-PE@TNF-Ab (S.C.) group, respectively. In addition, when compared with intraperitoneal administration, oral Casein-PE@TNF-Ab showed a slightly greater reduction in neutrophil infiltration (Supplementary Fig. 8).

## 4. Discussion

Recent studies have highlighted the potential of Pickering emulsions as an oral drug delivery platform for intestinal disease therapies, owing to their exceptional interfacial stability and ability to protect bioactive agents from harsh GI tract conditions (Niu et al., 2025; Hu et al., 2025; Sun et al., 2025). Oral delivery of TNF-Ab presents significant clinical advantages, including improved patient compliance, reduced administration costs, and localized therapeutic action that minimizes systemic exposure and associated side effects(de Silva et al., 2010). Despite these benefits, the oral delivery of TNF-Ab remains hindered by its vulnerability to enzymatic degradation in the GI tract and limited residence time in the colon, both of which compromise therapeutic efficacy. To overcome these challenges, we developed a casein-stabilized Pickering emulsion system to enhance the stability and colonic retention of TNF-Ab, thereby improving its therapeutic performance in the treatment of ulcerative colitis.

In this study, the concentration of casein and the water-to-oil ratio were optimized to formulate a stable and efficient Casein-stabilized Pickering emulsion (Casein-PE) for oral antibody delivery. As shown in Fig. 1, the optimal formulation was achieved using a 12:1 water-to-oil ratio and 6 mg/mL casein, resulting in a uniform particle size of 283.4 ± 4.0 nm, a strongly negative zeta potential of − 45.8 ± 0.6 mV, and a high encapsulation efficiency of 83.5 ± 0.9%. These physicochemical properties indicate a well-dispersed and electrostatically stable emulsion system. Importantly, the high encapsulation efficiency reflects the strong affinity between casein and the TNF-Ab at the oil–water interface, likely due to both electrostatic and hydrophobic interactions. Similar trends were observed in our previous work on protein-stabilized emulsions for antigen delivery, where optimal stabilizer concentration and water-to-oil ratio were 4 mg/mL and 12:1, respectively (Xie et al., 2025).

Given that the objective of this study was to achieve colonic delivery of TNF-Ab via oral administration, it was essential to evaluate the physicochemical stability of the formulation under simulated GI tract conditions, including SGF and SIF. In SGF, a notable increase in the particle size of Casein-PE-TNF-Ab was observed, which is likely attributable to acid- and pepsin-induced coagulation of casein micelles under gastric conditions(Ye et al., 2016) (Boirie et al., 1997). Although this increase in size may indicate structural changes, it could also serve as a protective mechanism by minimizing the premature release of TNF-Ab in the stomach. This protective effect was further supported by in vitro release studies, which showed that the release of TNF-Ab from Casein-PE was significantly slower than that of free TNF-Ab in SGF. Moreover, controlled release in the intestinal environment is advantageous for enhancing drug residence time and therapeutic exposure at the target site. Release profiles in SIF demonstrated a sustained release of TNF-Ab from Casein-PE@TNF-Ab, indicating its potential to achieve prolonged local delivery in the intestine. In addition, SDS-PAGE analysis confirmed that Casein-PE@TNF-Ab effectively protected the antibody from enzymatic degradation by pancreatin, highlighting its capacity to preserve protein integrity during intestinal transit. Consistent with these in vitro findings, the *in vivo* biodistribution demonstrated that Casein-PE protected the antibody from gastric degradation and markedly prolonged its residence in the colons of mice with DSS-induced colitis. This prolonged, localized exposure to the inflamed mucosa is expected to enhance TNF-α target engagement and therapeutic efficacy while potentially reducing systemic exposure and off-target effects.

These data indicate that Casein-PE@TNF-Ab accumulates in the colon. We therefore tested whether this translates into improved therapeutic efficacy compared with free antibody. In our study, in vivo results demonstrated that oral administration of Casein-PE@TNF-Ab significantly increased colon length compared to oral administration of the free antibody (Fig. 6C–D), indicating effective alleviation of colonic tissue damage. This therapeutic benefit may be attributed to the controlled release behavior of the formulation—Casein-PE@TNF-Ab exhibited delayed release in SGF, sustained release in intestinal fluid, and enhanced protection of the antibody from enzymatic degradation by pancreatin. Given that TNF-Ab is conventionally delivered via injection in clinical practice, we further compared the oral Casein-PE@TNF-Ab with S.C. injection of the same formulation. Remarkably, oral administration resulted in superior therapeutic efficacy compared to the S.C. route. This improvement may be due to the pathological characteristics of ulcerative colitis (UC), which involve epithelial barrier disruption and localized inflammation in the colon (Xiao et al., 2016). Therefore, direct delivery of TNF-Ab to the inflamed colonic tissue via the oral route allows for higher local drug concentrations, enhanced targeting of the inflammatory microenvironment, and reduced systemic exposure—collectively contributing to improved therapeutic efficacy. Compared to previously reported nanoparticle-based oral delivery systems for TNF-Ab in IBD models(Shrestha et al., 2022), which showed therapeutic efficacy but did not result in a significant improvement in colon length relative to the DSS group, our formulation demonstrated a comparable trend in colonic protection.

In addition to the restoration of colon length, oral administration of Casein-PE@TNF-Ab significantly downregulated the expression of key inflammatory mediators, including TNF-α, IL-1β, and MPO, as well as the infiltration of neutrophils into colonic tissue. The therapeutic mechanisms of TNF-Ab in UC are generally attributed to two distinct yet complementary pathways: (1) neutralization of soluble TNF-α (sTNF-α), and (2) interaction with transmembrane TNF-α (tmTNF-α), both of which contribute to suppression of inflammation and modulation of the immune response (Silva et al., 2010). The reduction in colonic TNF-α expression suggests that TNF-Ab effectively neutralized sTNF-α, thereby interrupting the pro-inflammatory feedback loop and limiting further cytokine production. Additionally, TNF-Ab may engage tmTNF-α expressed on immune cells and initiate reverse signaling, a process known to inhibit the release of inflammatory cytokines such as IL-1β. However, some studies have reported that under LPS stimulation, reverse signaling induced by agents such as etanercept does not suppress the LPS-induced release of IL-1β, suggesting that the anti-inflammatory effects of tmTNF-α engagement may be context-dependent (Kirchner et al., 2004). Moreover, TNF-α has been shown to activate vascular endothelial cells and induce the expression of neutrophil chemotactic factors in a time- and concentration-dependent manner (Strieter et al., 1989). This chemotactic signaling promotes neutrophil recruitment and transmigration into inflamed colonic tissues, thereby exacerbating mucosal inflammation. These neutrophils, once recruited, release MPO, a key enzyme that amplifies oxidative stress and tissue damage, thereby exacerbating mucosal inflammation. Consequently, TNF-Ab–mediated suppression of TNF-α may indirectly reduce neutrophil recruitment and subsequent MPO release, further contributing to the resolution of intestinal inflammation. Similar findings have been reported in clinical studies, where anti-TNF-α therapy was shown to inhibit the production of neutrophil-derived proinflammatory mediators in patients with inflammatory bowel disease (Zhang et al., 2018). In addition, while previous studies relied on parenteral administration, our study demonstrated that oral delivery of Casein-PE@TNF-Ab effectively suppressed both neutrophil infiltration and MPO expression, thereby offering a non-invasive strategy with comparable therapeutic efficacy in the treatment of UC.

Our strategy effectively delivered TNF-Ab via the oral route for the treatment of DSS-induced colitis in mice, demonstrating its promise as a viable oral delivery platform for biologics in intestinal disease therapy. However, the stability of Casein-PE@TNF-Ab in the gastric environment requires further improvement to enhance its protective capacity. In addition, for broader clinical translation, long-term studies are needed to evaluate its safety, biocompatibility, and potential immunogenicity. Future research may focus on further engineering the Pickering emulsion interface, such as by incorporating enteric coatings or stimuli-responsive materials, to achieve precise site-specific release in the inflamed colon. Moreover, the platform’s adaptability to load other therapeutic proteins or antibodies offers opportunities for expanding its application to a wide range of gastrointestinal disorders. From a translational perspective, the Casein-PE system may also be advantageous due to its simple composition and straightforward preparation process, which are compatible with conventional industrial emulsification technologies. In addition, the use of biocompatible and relatively low-cost materials such as casein and squalene may further facilitate large-scale production. These characteristics suggest that the platform has potential feasibility for scale-up manufacturing and future clinical application. Ultimately, such oral delivery systems could serve as patient-friendly alternatives to parenteral administration, reducing treatment burden while maintaining or enhancing therapeutic efficacy. Together, these findings demonstrate that Casein-PE@TNF-Ab not only mitigates inflammatory damage through multiple immunological pathways but also underscores the potential of this oral delivery platform for targeted antibody-based therapy in colonic inflammatory diseases.

## 5. Conclusion

In this study, we developed a casein-stabilized Pickering emulsion for oral delivery of anti-TNF-α antibody. The system protects the payload from gastric degradation and prolongs its residence in the colon in a DSS-induced mouse model of ulcerative colitis. The optimized formulation remained stable at 4 °C for at least three months, with a uniform milky appearance and essentially unchanged particle size and Zeta potential while preserving bioactivity. After 3 months storage, the formulation loaded with TNF-Ab maintained bioactivity and effectively neutralized TNF-α. Oral Casein-PE@TNF-Ab increased antibody retention and local exposure at the colon and provided therapeutic benefit, as evidenced by longer colon length and attenuated mucosal inflammation, including lower tissue TNF-α, IL-1β, and MPO, together with reduced neutrophil infiltration. These findings support casein-stabilized Pickering emulsions as a practical and noninvasive platform for oral delivery of therapeutic antibodies in inflammatory bowel disease, with clear translational potential.

## Supplementary Material

Supplement Figures

## Data Availability

Data will be made available on request.

## Appendix A. Supplementary data

Supplementary data to this article can be found online at https://doi.org/10.1016/j.ijpharm.2026.126881.
