# Supplementary material for: Pickering emulsion as an oral delivery platform of anti-TNF-α antibody for ulcerative colitis therapy
Source: Int J Pharm. Author manuscript; Available in PMC 2026 Apr 25. (PMC13107477; doi:10.1016/j.ijpharm.2026.126881)
Supplement: Supplement Figures [file NIHMS2166863-supplement-Supplement_Figures.pdf]

Supplementary Figure 1 The FT-IR spectrum of Free Casein, TNF-Ab and Casein@PE-TNF-Ab

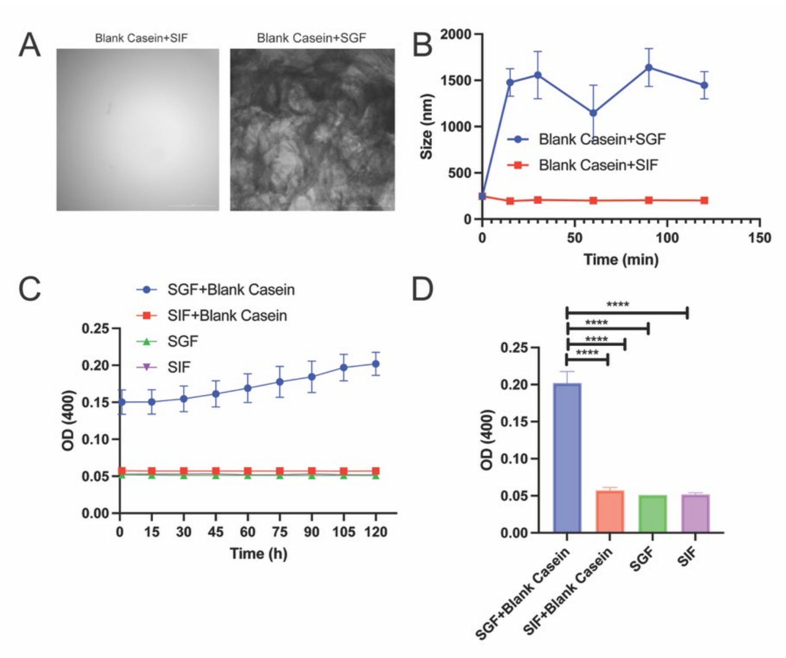

Supplementary Figure 2 Microscopic images (A), particle size distribution (B), and optical density (OD) values (C–D) of blank casein after incubation with SGF and SIF. Data are presented as mean ± SEM (n = 3). Statistical analysis was performed using one-way ANOVA. $^{****}P < 0.0001$.

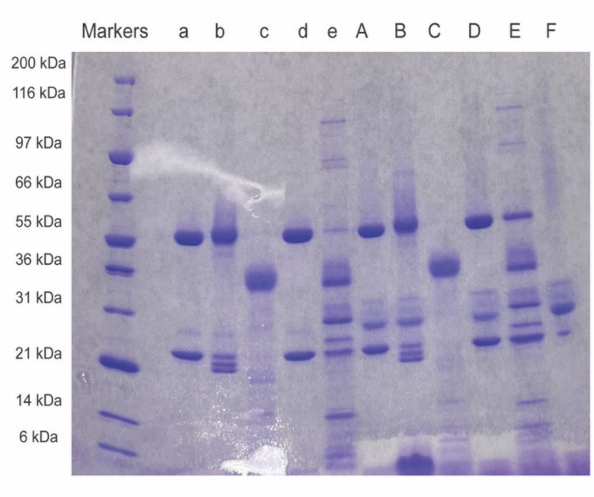

Supplementary Figure 3 SDS-PAGE analysis of free TNF-Ab and Casein-PE@TNF-Ab after incubation in SGF (with or without pepsin) and SIF (with or without pancreatin), a: free TNF-Ab control, b: free TNF-Ab SGF without pepsin, c: free TNF-Ab SGF with pepsin, d: free TNF-Ab in SIF without pancreatin, e: free TNF-Ab in SIF with pancreatin, A: Casein-PE@TNF-Ab control, B: Casein-PE@TNF-Ab SGF without pepsin, C: Casein-PE@TNF-Ab SGF with pepsin, D: Casein-PE@TNF-Ab in SIF without pancreatin, E: Casein-PE@TNF-Ab in SIF with pancreatin, F: blank Casein-PE.

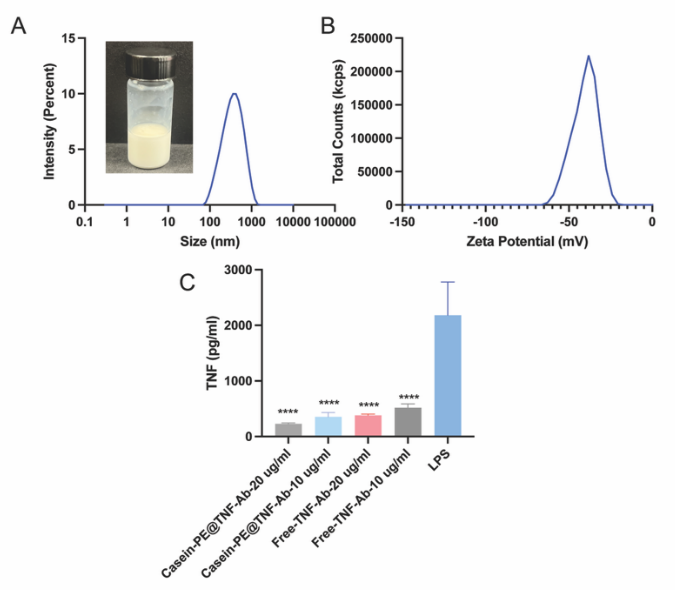

Supplementary Figure 4 Three-month stability of Casein-PE at 4 °C and retention of bioactivity. (A) Particle size and visual appearance after storage. (B) Zeta potential. (C) Neutralization of TNF-α by Casein@PE-TNF-Ab in LPS-stimulated RAW264.7 cells. $^{****}P <$ 0.0001, (mean ± SD n = 4). Data were analyzed by one-way ANOVA.

	Particle Size (nm)	PDI	Zeta Potential (mV)
Casein-PE@TNF-ab	337.3±6.6	0.211±0.014	-52.4±1.07
Casein-PE@OVA-647	326.2±0.6	0.215±0.039	-45.7±1.2
Casein-PE@OVA-Cy7	324.9±1.1	0.223±0.016	-52.0±2.2

Supplementary Table 1 The particle size and Zeta potential and Zeta Potential of Casein-PE-OVA-647 and Casein-PE-OVA-Cy7

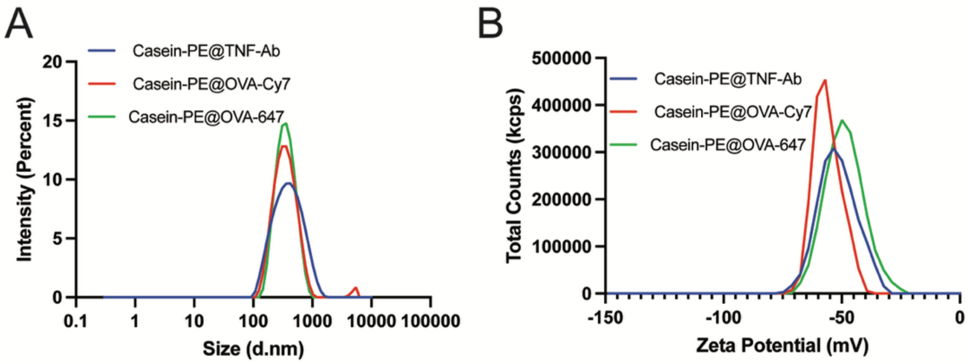

Supplementary Figure 5 Particle size (A) and zeta potential (B) of Casein-PE@OVA-647 and Casein-PE@OVA-Cy7.

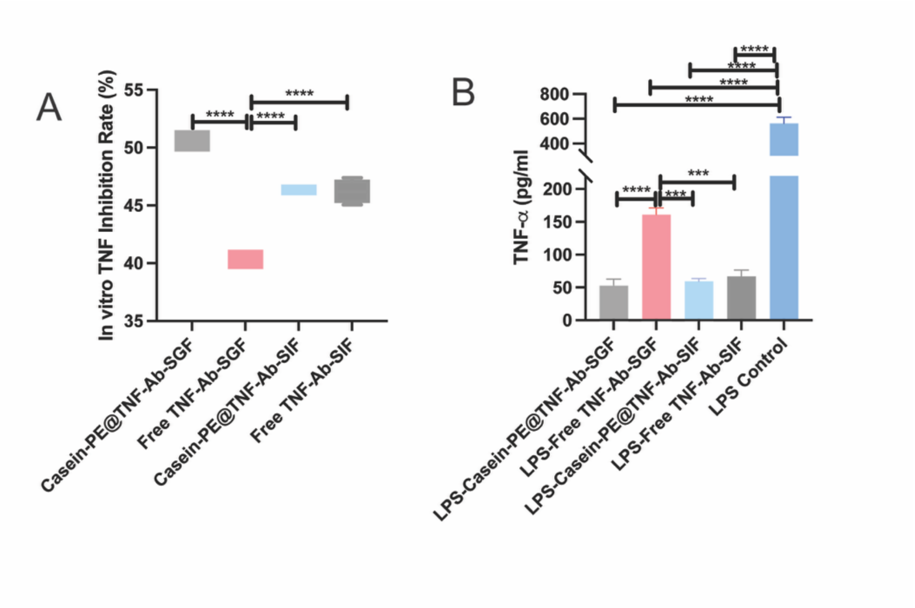

Supplementary Figure 6 (A) In vitro TNF-α inhibition by Casein-PE@TNF-Ab and free TNF-Ab after SGF and SIF incubation. (B) TNF-α neutralization in LPS-stimulated RAW264.7 cells by Casein-PE@TNF-Ab and free TNF-Ab following SGF and SIF incubation $^{***}P < 0.001$, $^{****}P < 0.0001$. n = 3, data are presented as mean ± SEM. Data were analyzed by one-way ANOVA.

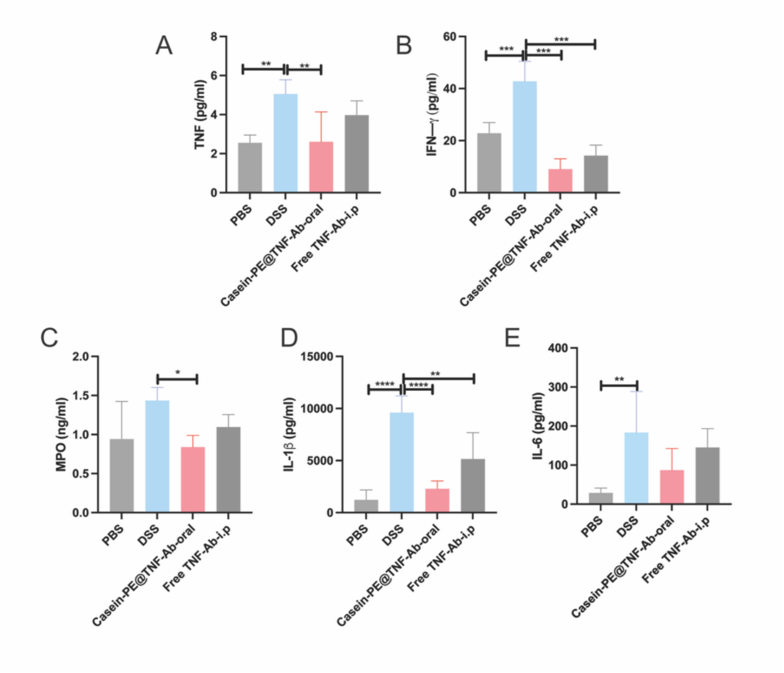

Supplementary Figure 7 Expression of TNF-α (A), IFN-γ (B), MPO (C), IL-1β (D) and IL-6 (E) in colon tissues following treatment with oral Casein-PE or intraperitoneal (i.p.) free TNF-Ab. $^{*}P < 0.05$, $^{**}P < 0.01$, $^{***}P < 0.001$, $^{****}P < 0.0001$. n = 5, data are presented as mean ± SEM. Data were analyzed by one-way ANOVA.

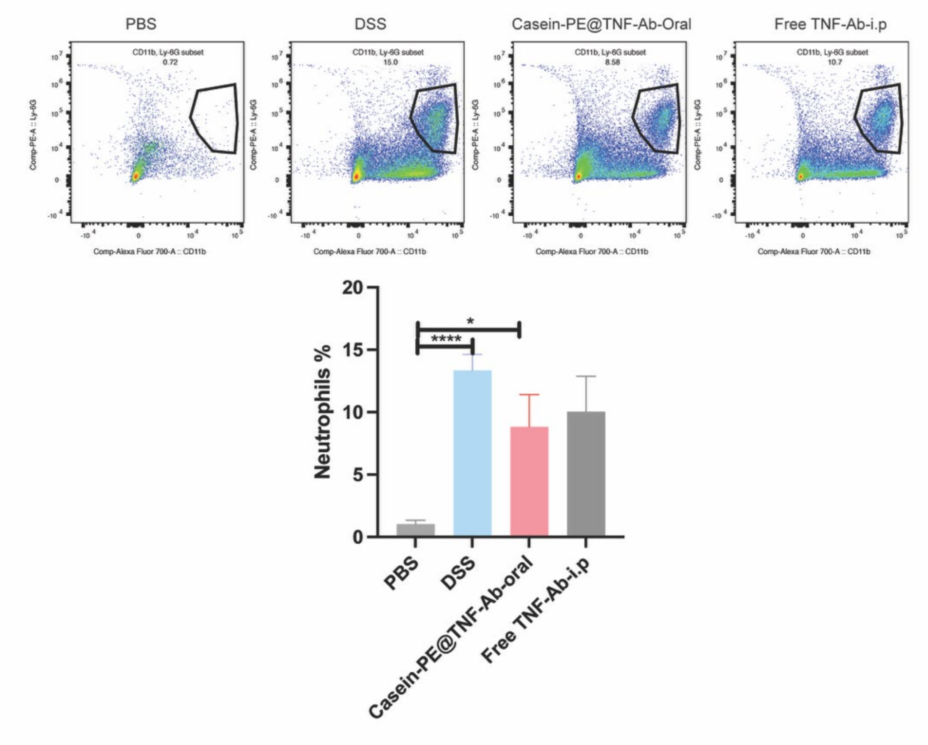

Supplementary Figure 8 Representative flow cytometry dot plots and quantitative analysis of neutrophil populations in colon tissues after treatment with oral Casein-PE and i.p. free TNF-Ab. $^{*}P < 0.05$, $^{***}P < 0.001$. n = 5, data are presented as mean ± SEM. Data were analyzed by one-way ANOVA.
